# Acoustic Analysis of Primary Care Patient-provider Conversations to Screen for Cognitive Impairment

**DOI:** 10.64898/2025.12.27.25343088

**Published:** 2025-12-29

**Authors:** Joseph T. Colonel, Jacqueline Becker, Lili Chan, Cara Faherty, Tielman van Vleck, Laura Curtis, Juan Wisnivesky, Alex Federman, Baihan Lin

**Affiliations:** 1 Department of Psychiatry, Icahn School of Medicine at Mount Sinai, New York, NY, USA; 2 Division of General Internal Medicine, Icahn School of Medicine at Mount Sinai, New York, NY, USA; 3 Institute for Personalized Medicine, Icahn School of Medicine at Mount Sinai, New York, NY, USA; 4 Division of General Internal Medicine, Feinberg School of Medicine at Northwestern University, Chicago, IL, USA

## Abstract

**Importance:**

Cognitive impairment (CI) is often under detected in primary care due to time and resource constraints. Passive analysis of clinical dialogue may offer an accessible approach for screening.

**Objective:**

To assess whether audio recordings of patient–physician dialogue during routine primary care visits can be used to identify CI using acoustic speech features and machine learning (ML).

**Design:**

This observational study conducted among older primary care patients involved audio recording primary care visits using a microphone and portable device. An external validation cohort was recruited in a separate city to assess reproducibility of findings.

**Setting:**

The study was conducted in primary care practices in New York City, with additional participants recruited from primary care practices in Chicago, Illinois, for validation.

**Participants:**

The study included 787 English-speaking patients aged 55 years and older, without documented history of dementia or mild CI. Eligible patients were recruited from primary care practices during routine visits. For validation, 179 patients meeting the same eligibility criteria were recruited from primary care practices in Chicago.

**Exposures:**

Multiple thirty-second speech segments were extracted from recordings. Acoustic features were derived using foundation models (Whisper, HuBERT, Wav2Vec 2.0) and expert-defined methods (eGeMAPS, prosody).

**Main Outcomes and Measures:**

CI was defined as Montreal Cognitive Assessment score ≥1.0 standard deviations below age and education-adjusted norms. ML classifiers were trained to predict CI status from audio recordings. We calculated area under the receiver operating characteristic curve (AUC-ROC) and maximum F1 score (F_max_) for identifying CI participants.

**Results:**

The mean age was 66.8 years and 21% had CI. Models using Whisper-derived acoustic features performed best (AUC-ROC=0.733, 95% confidence interval [95%CI]=0.714-0.752; F_max(CI)_=0.504, 95%CI=0.474-0.534). Results generalized to the external site with similar performance (AUC-ROC=0.727, 95%CI=0.714-0.740; F_max(CI)_=0.459, 95%CI=0.442-0.476). Model interpretation identified pitch, timing, and variability features as key predictors. When used for screening, the algorithm achieved positive predictive value of 30.4% (95%CI=28.7%-32.1%), sensitivity of 68.2% (95%CI=61.8%-74.6%), and specificity of 63.6% (95%CI=59.8%-67.4%) on the holdout cohort.

**Conclusions and Relevance:**

ML models trained on acoustic features from brief clinical conversations identified CI with high accuracy. These findings support the feasibility of passive, speech-based screening during routine primary care.

## Introduction

Cognitive impairment (CI) affects nearly 1 in 5 adults in the United States aged 65 years and older,^1^ yet it often goes unrecognized by clinicians until it reaches advanced stages.^1–8^ It is also less likely to be recognized in minoritized older adults, potentially furthering disparities in health care delivery and outcomes.^9^ The United States Preventive Services Task Force has found insufficient evidence to assess the balance of benefits and harms for early detection of CI through screening.^10^ Nevertheless, there are various reasons for screening and early detection, including identifying and addressing reversible causes of CI, reducing risks of harm that may be caused by medications, addressing co-morbid conditions (e.g., depression) that may worsen cognitive functioning, improving support for caregivers to alleviate the stress and burden associated with caring for people with CI,^11–19^ and improving self-management support^20^ and coordination of care.^18,21^ Additionally, new treatments for Alzheimer’s Disease (AD) have recently emerged that can slow cognitive decline in dementia and the benefits may be greatest for those treated earlier in the disease course.^22–23^

Primary care is the most appropriate setting for dementia screening.^1,4,7,24^ However, routine dementia screening occurs infrequently in primary care despite the availability of brief assessments like the Mini-Mental State Examination (MMSE) and the Montreal Cognitive Assessment (MoCA).^2,5,25^ Overall, fewer than one-third of older adults are ever screened for CI.^1,2,26^ Instead, primary care providers typically assess cognitive functioning when patients present with a specific cognitive complaint. Moreover, they miss CI in up to 76% of patients when using routine data gathering (e.g., history, physical examination).^5^ Competing priorities for primary care physicians, like providing routine preventive care, may contribute to the low rates of CI screening in primary care settings.

To overcome some of the barriers to screening, researchers have worked to develop automated CI detection using machine and deep learning models applied to medical record data.^27–31^ Such models have performed well for identifying patients with known CI,^32^ but are not accurately performing in screening for new cases.^28^ A promising new avenue for automated CI detection is the analysis of speech, which is increasingly recognized as a clinical biomarker of cognitive functioning.^33–34^ Low-cost, high-quality voice recorders saturate the market,^35^ greatly expanding the feasibility of speech-based CI screening in clinical settings.

Beyond laboratory-based assessments and electronic health record approaches, there has been growing interest in moving CI prediction closer to the point of care. Prior studies have explored the use of short, structured speech tasks (e.g., picture description) administered in research settings to train machine learning models for Alzheimer’s disease and MCI detection.^36^ While these approaches demonstrate strong diagnostic accuracy, they often require specialized test administration, standardized recording environments, and substantial participant time, which limit scalability and generalizability. In contrast, fewer studies have examined speech generated during naturalistic clinical encounters.^37^

While these and other studies demonstrate proof of concept, their application to CI screening in primary care is limited by design. Specifically, none were designed to identify individuals in primary care with no prior history of CI. In addition, embedding speech-based CI screening into existing care processes could overcome key barriers of cost, time, and accessibility, while capturing ecologically valid representations of patients’ everyday communicative and cognitive functioning. Our study extends beyond controlled laboratory contexts by evaluating speech from real-world primary care encounters. We recruited patients without known CI, administered a validated cognitive screener as a gold standard, and developed machine learning algorithms for CI screening in primary care settings.

## Methods

### Subjects and Settings

Patients were recruited from primary care practices in the Mount Sinai Health System in New York City, NY and the Northwestern Memorial Hospital in Chicago, IL from August 2020 through December 2021. The practices included three hospital-based teaching clinics and two multi-provider faculty clinics. We included patients attending a routine primary care visit who were ages 55 and older and spoke English. Patients with a diagnosis of MCI, dementia or related disorders in the problem list or medical history of their medical record were excluded (ICD10-CN codes G20, G30, G31, G91, F00-F03, F09, R41, R47, I69). Study procedures were approved by the Institutional Review Boards of the Icahn School of Medicine at Mount Sinai and the Northwestern University Feinberg School of Medicine.

### Recruitment and Interviews

Research coordinators (RC) screened a list of potentially eligible patients scheduled for a routine primary care visit generated from the health systems’ electronic medical records and obtained permission of the patients’ primary care providers to recruit them for study participation. A recruitment letter was then mailed to a random selection of individuals followed by a telephone call, during which the RC described the study and administered an eligibility screener. Subjects meeting final eligibility criteria were invited for an in-person interview scheduled to immediately follow their upcoming primary care appointment. The 30-minute interviews were conducted by trained RCs in an exam room.

### Audio Data Collection

Participants were recorded during their scheduled visits with primary care physicians using a Tascam DR-10L Micro Portable Audio Recorder with lavalier attached to a lapel or collar at a 44.1 kHz sampling rate, stored as 16-bit linear pulse code modulation (PCM) waveform audio (WAV) files.

### Gold Standard Assessment of Cognitive Impairment

We used the MoCA as the gold standard assessment of CI because it is widely used for dementia screening.^9, 38–40^ It consists of 12 tasks covering visuospatial/executive functioning, naming, memory, attention, language, delayed recall and orientation. Scores range from 0-30. Raw scores were converted to age and education adjusted z-scores. We then defined CI as MoCA scores ≥1.0 standard deviation below the mean of normative data.^39,41–44^ A detailed analysis of CI in the study cohort is described elsewhere.^3^

Data were also collected on standard sociodemographic characteristics of study participants, including age, gender, race and ethnicity, English language proficiency (ELP) and country of birth. ELP was assessed with a single item, “How would you describe your ability to speak and understand English?” with 6 response options ranging from very poor to excellent. Low ELP was defined as a response of very poor, poor or fair.

## Acoustic Methods

### Acoustic Preprocessing

We evaluated several preprocessing pipelines to assess how acoustic processing influences classification performance and model generalizability. Analyses were conducted on two audio types: recordings containing both patient and clinician speech, and recordings containing only patient speech.

For the combined audio, we tested untreated recordings and versions processed with voice activity detection (VAD) to reduce noise using Silero VAD.^45^ VAD was applied to the full-length recordings to identify dialog passages. To control potentially confounding acoustic noise, all audio segments with no detected voice were silenced. All others were unaltered to preserve the dialog’s acoustic characteristics.

For patient-only audio, we applied the Cocktail Fork Separation (CFS) algorithm to isolate the patient’s voice and silence background noise and clinician speech.^46^

### Acoustic Feature Extraction

We extracted five sets of acoustic features that capture foundation model, paralinguistic, and prosodic information from patient speech. Deep neural network (DNN) feature extraction was performed on mono audio downsampled to 16 kHz for compatibility with pre-trained models (Whisper,^47–48^ HuBERT,^49–50^ Wav2Vec2.0^51–52^) by extracting and averaging final-layer embeddings. Expert-defined feature extraction (eGeMAPS,^53–55^ prosody^56–58^) was performed on the 44.1kHz mono waveform. Features were extracted on non-overlapping 30 second segments of each recording. Full details regarding each featureset can be found in the Supplementary materials.

## Machine Learning Methods

### Machine Learning Setup

**Figure 1.**
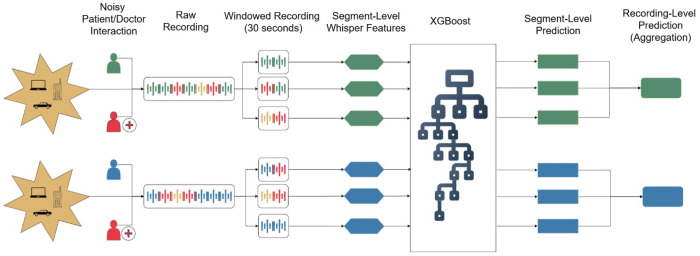
Acoustic preprocessing, feature extraction, and classification pipeline

To predict CI from spontaneous patient speech, we implemented a two-stage machine learning framework: (1) segment-level classification of speech excerpts, and (2) participant-level aggregation of segment predictions. The design was optimized for robustness to class imbalance and interpretability in clinical contexts. A diagram of our full pipeline is shown in Figure 1.

### Segment-Level Processing

Each participant’s speech was divided into fixed, non-overlapping 30-second segments. All acoustic features (Whisper, HuBERT, W2V2, eGeMAPS, and prosody) were extracted per segment. Feature vectors were z-normalized using statistics computed on the training folds during cross-validation.

### Segment-Level Classification

We trained an XGBoost classifier to predict whether each segment was associated with CI.^59^ One classifier was trained per feature set. Due to class imbalance at the participant level, we applied random oversampling of the minority class (cognitively impaired participants) in the training set. Oversampling was performed at the participant level, duplicating all segments from selected participants to preserve intra-subject structure and avoid data leakage.

### Participant-Level Aggregation

Participant-level predictions were generated by aggregating segment-level impairment probabilities. We evaluated several aggregation strategies including mean, median, and BioBERT inspired aggregation,^60^ where we upweighted the segment with the highest predicted probability by a factor of c relative to the mean of the remaining segments. We evaluated values of c ∈ {1, 2, …, 9}, optimizing c via cross-validation.

### Model Evaluation

Model performance was evaluated using k-fold cross-validation stratified by CI prevalence at the participant level to ensure no leakage of speech content across folds. We report Area Under the ROC Curve (AUC-ROC) and F_max_ of the CI class (F_max(CI)_),^61^ which is defined as maximum F1 score achieved across all probability thresholds, computed specifically for the CI class. All metrics were averaged across folds.

To evaluate generalizability, we assessed model performance on an external holdout dataset collected at Northwestern Memorial Hospital clinics using the same training folds derived from the Mount Sinai data.

### Positive Predictive Values for Clinical Application

To assess potential clinical applicability, we explored CI classifiers built using the Whisper-derived speech embeddings from the Mount Sinai cohort. Each model configuration corresponded to a different preprocessing pipeline and was evaluated using AUC-ROC to identify the best-performing classifier. This selected classifier formed the basis of a speech-based screening algorithm designed to flag individuals for formal cognitive assessment based on their recorded clinical dialogue.

For screening implementation, each patient’s dialogue recording was processed through the trained classifier, which produced a continuous output between 0 and 1 representing the predicted probability of CI at the end of the recording. A predefined threshold was then applied to this probability such that when the estimated probability of CI was higher than the threshold, the patient would be flagged for further CI screening. Three preprocessing pipelines were used, each requiring a distinct threshold (0.25 for the raw, 0.39 for the VAD, and 0.28 for the CFS audio).

Thresholds were chosen to prioritize positive predictive value (PPV), sensitivity, and specificity, as these measures are more clinically relevant than overall classification accuracy in screening contexts. Assuming a 20% prevalence of undiagnosed CI in primary care,^3^ consistent with prior estimates, we selected thresholds to achieve a target PPV of approximately 30%, balancing clinical utility with acceptable false-positive rates.

## Results

### Study Enrollment and Participant Characteristics

Among 3,815 potentially eligible persons, RCs contacted 2,894 (75.9%) by telephone; 1,737 (60.0%) declined study participation and 208 (7.2%) were unable to complete screening in English; 949 (32.8%) were eligible and agreed to study participation, 918 provided signed consent and 787 (20.1%) completed the in-person interview and had a primary care dialogue recorded.

The mean age of study participants was 66.8 years; 55% were male, 32.4% Black, and 25.7% were Hispanic (Table 1). The overall rate of undiagnosed CI based the MoCA was 20.7%.

### Collected Audio Data

At the Mount Sinai clinics, a total of 388.6 hours of audio were recorded, with mean [SD] primary care encounter duration of 29.6 [13.0] minutes. At the Northwestern clinics, a total of 70.6 hours of audio were recorded, with mean primary care encounter duration of 23.7 [9.7] minutes. Demographic characteristics of the study populations stratified by site are shown in Supplemental Table 1.

### Classification Performance by Acoustic Feature Set

**Figure 2.**
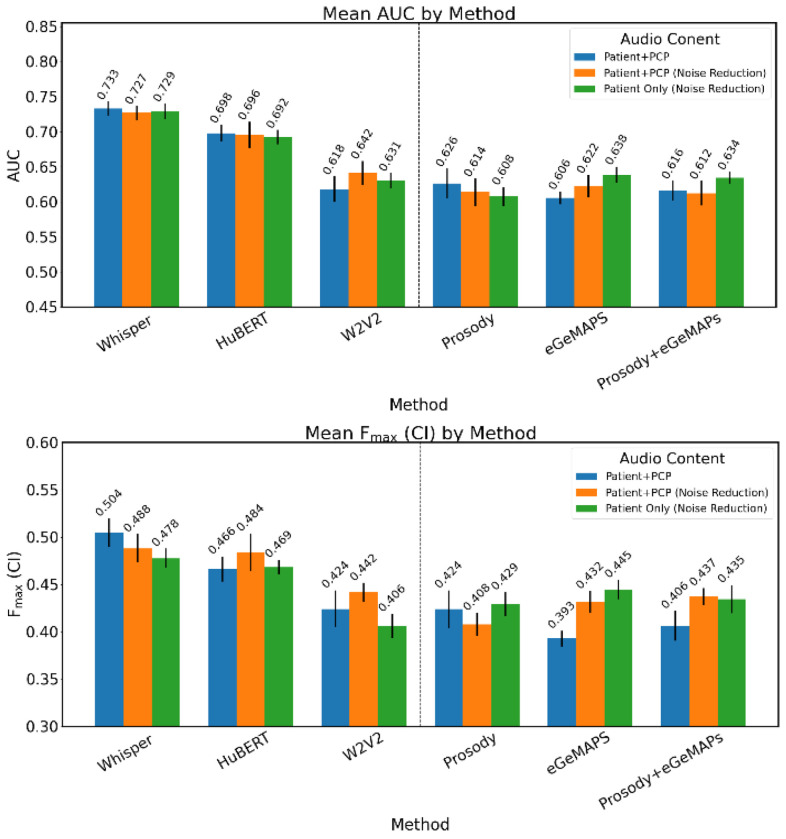
Classification performance on derivation dataset (N=787) n

Performance of the different models for classification in the derivation set are shown in Figure 2. The DNN-derived features outperformed the expert-defined features in the evaluation. Whisper embeddings achieved the highest overall classification performance, achieving a mean AUC-ROC of 0.733 (95% confidence interval [95% CI] = 0.714-0.752) and mean F_max(CI)_ of 0.502 (95% CI = 0.471-0.533) on the unmodified audio recordings, followed by HuBERT-derived and W2V2 within the DNN-based features (See Figure 2).

Models based on eGeMAPS and prosodic features showed lower but non-negligible performance. Of the two featuresets, eGeMAPS performed best on isolated patient audio, with mean AUC-ROC of 0.638 (95% CI = 0.615-0.661) and F_max(CI)_ of 0.445 (95% CI = 0.426-0.464), with the prosody features performing slightly better on the unmodified audio.

### External Validation

**Figure 3.**
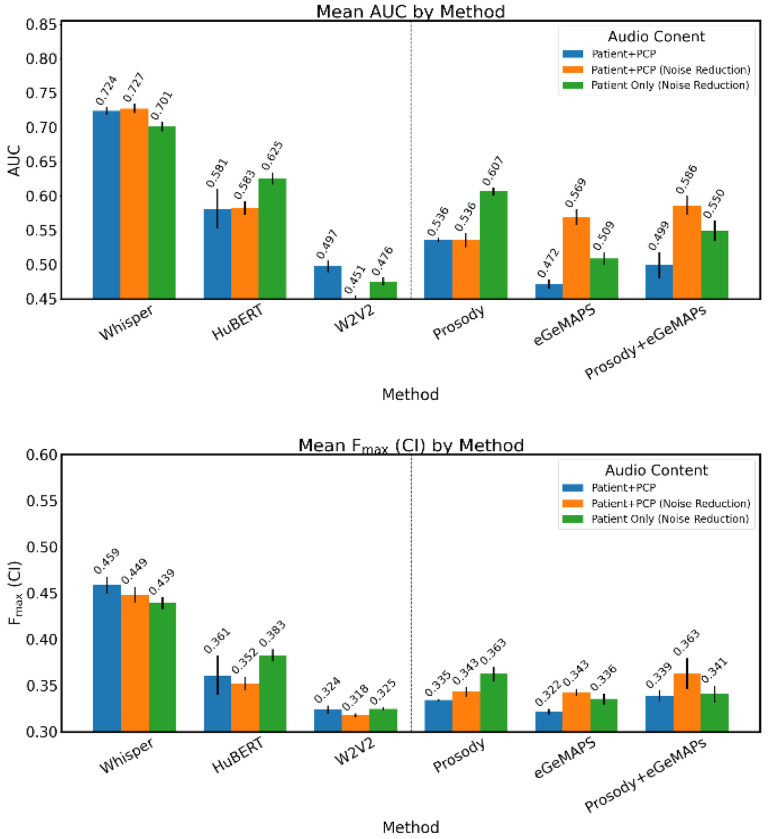
Classification Performance on evaluation dataset (N=179)

Results of the validation models are shown in Figure 3. Models trained on Whisper-derived features demonstrated the highest generalizability, maintaining comparable classification performance relative to internal testing. The best performing model achieved mean AUC-ROC of 0.727 (95% CI = 0.714-0.740) on noise-reduced dialogue audio and F_max(CI)_ of 0.459 (95% CI = 0.441-0.477) on untreated audio. Similarly, models utilizing prosodic features from patient-only audio segments showed stable performance, with mean AUC-ROC of 0.607 (95% CI = 0.596-0.618) and F_max(CI)_ of 0.363 (95% CI = 0.349-0.377). In contrast, all other models had diminished predictive accuracy when applied to the external dataset.

### Screening Algorithm

The Whisper model employing data with noise reduction preprocessing had the best performance for screening, with sensitivity of 83.4% (95% CI = 79.2%-87.6%) and specificity of 44.4% (95% CI = 42.1%-46.7%) in the derivation set (at PPV 28.2% (95% CI = 26.4%-29.6%)) and sensitivity of 68.2% (95% CI = 61.8%-74.6%) and specificity of 63.6% (95% CI = 59.8%-67.4%) in the validation set (at PPV 30.4% (95% CI = 28.7%-32.1%)) (Table 2). Sensitivity was lower in the validation set with raw audio and CFS preprocessing.

### Model Interpretation

**Figure 4.**
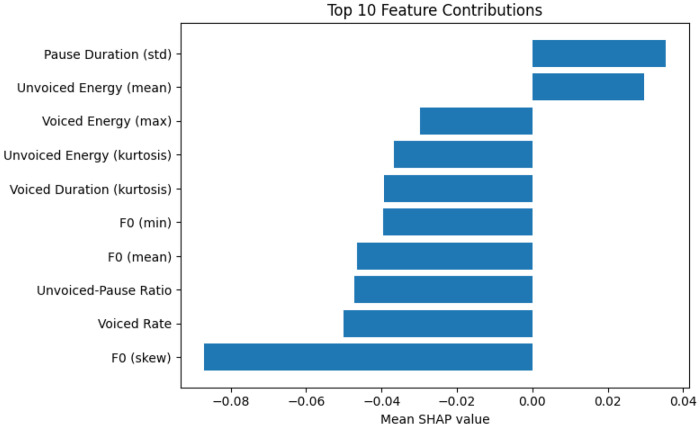
SHAP interpretation analysis of prosody featureset on Northwestern holdout data

Model interpretability analyses provided insights into the specific speech characteristics associated with CI. Using SHAP (Shapley Additive Explanations)^62^ values applied to the XGBoost models trained on prosodic features derived from patient-only audio, the top 10 influential features for prediction are plotted in Figure 4. Increased energy in unvoiced segments and greater variability in pause duration were associated with higher likelihood of predicting CI. In contrast, higher values of features related to fundamental frequency (F0), voicing rate, and energy dynamics were more indicative of predicting healthy cognition.

## Discussion

We developed and evaluated machine learning models using acoustic foundation model embeddings derived from primary care dialogue. Among several architectures tested, models based on Whisper embeddings achieved the highest predictive accuracy, with mean AUC-ROC values exceeding 0.72 in a holdout dataset. After setting a screening cutoff threshold for the output of the classification model, the algorithm yielded sensitivity of 68.2% and specificity of 63.6% in the validation set (at PPV 30.4%). Preprocessing techniques that reduced ambient noise while preserving conversational integrity yielded the most robust performance on holdout data. These findings highlight both the feasibility and technical promise of a scalable, speech-based cognitive assessment for primary care. By leveraging naturally occurring speech from primary care visits, and without adding burden to clinical workflows, our approach could complement existing screening strategies and support earlier detection of CI in real-world care settings.

To our knowledge, this is the first study to conduct passive screening for undiagnosed CI from recorded patient-clinician dialog, and the classification performance aligns with screening literature using other stimuli. For example, Schäfer et al ^31^ achieved AUC-ROC of 0.73-0.77 on intra-cohort CI screening when evaluating a speech-based biomarker for CI. In contrast to our assessment of cognition under naturalistic conditions, the Schäfer study used a mobile recording platform that recorded participants undergoing formal cognitive assessments like semantic verbal fluency. Our findings highlight the potential of unstructured conversational data as a novel and complementary modality for passive CI screening.

The generalizability of the Whisper-based models, as evidenced by their consistent performance on the holdout data, may be due to the diverse acoustic conditions and speaker demographics seen in the web-scale, multilingual dataset used to train the foundation model. In comparison, HuBERT and W2V2 were primarily trained on datasets of English-language audiobook recordings. Similarly, prosodic features may have outperformed the eGeMAPS features as prosodic measures are more robust to varying recording environments than eGeMAPS features.^63^

These findings suggest that features from speech foundation models may capture clinically relevant patterns more effectively than traditional acoustic features. This observation aligns with state-of-the-art acoustic approaches for CI classification from structured laboratory assessments, where winners of the PROCESS^36^ and TAUKADIAL challenges^64^ used Whisper-derived acoustic features for classification.^48,65^

The tradeoff of using DNN-based features is reduced interpretability. Alternatively, expert-defined features, though underperforming, provide interpretable insights into physiological changes associated with CI since they explicitly derive from speech production processes. Therefore, we performed interpretation analysis on the best expert-defined feature model. These findings suggest that disrupted speech timing, reduced vocal dynamism, and sex-related pitch characteristics contribute to the acoustic profile of CI. This agrees with previous findings showing pitch-related prosody features vary significantly with CI in Parkinson’s disease.^66^ Other meta-analyses have highlighted that pause-related features and rate of speech vary significantly between CI and control populations.^67–68^

### Limitations

This study has several limitations. Although recordings were collected during routine care, all data were collected in two affiliated clinical sites using a uniform protocol, which may limit generalizability. Second, while the models capture differences in acoustic features, they do not account for lexical content, which may provide complementary diagnostic value. Third, the inclusion of only English speakers may limit the applicability of this approach to non-English speaking populations.

### Conclusion

This study shows that analyzing the acoustic patterns of ordinary patient–clinician conversations could help fill an important gap in cognitive screening. Machine learning models trained on acoustic features from brief clinical conversations identified CI with high performance in-line with the CI screening literature. These findings support the feasibility of passive, speech-based screening during routine primary care. Future work should validate these findings in larger and more diverse populations, assess longitudinal predictive utility, and explore integration with existing electronic health record data. Ultimately, speech-based screening tools may offer a noninvasive, low-cost approach to identifying CI earlier and more equitably across clinical populations.

## Supplementary Material

1

## Figures and Tables

**Table 2.** Performance of Screener on Derivation and Validation datasets ± 95% Confidence

Preprocessing	AUC-ROC	Positive Predictive Value	Sensitivity	Specificity
Raw Audio				
Derivation set	0.733±0.020	31.1±2.3%	82.1±4.5%	52.4±3.1%
Validation set	0.724±0.011	30.4±0.9%	51.7±7.4%	72.6±3.0%
Noise Reduction				
Derivation set	0.727±0.019	28.2±1.8%	83.4±4.2%	44.4±2.3%
Validation set	0.727±0.013	30.4±1.7%	68.2±6.4%	63.6±3.8%
Source Separation				
Derivation set	0.729±0.021	30.7±1.6%	83.9±4.2%	50.6±2.0%
Validation set	0.701±0.014	30.4±1.8%	49.4±4.2%	73.6±2.4%

## Key Points

### Question:

Can acoustic features extracted from audio recordings of patient–physician conversations during routine primary care visits be used to screen for cognitive impairment?

### Findings:

In this study including 787 older adults without diagnosis of cognitive problems, machine learning models trained on acoustic features from audio segments of recordings of primary care visits achieved area under the receiver operating characteristic curve values of 0.72 for predicting cognitive impairment. The algorithm achieved a sensitivity of 83%, specificity of 44%, and positive predictive value of 28%, identifying a subset of primary care patients at higher risk for cognitive impairment. Models performed similarly on an external validation dataset of 179 participants. Interpretability analyses highlighted patient pause duration and energy-related features as salient indicators of cognition status.

### Meaning:

These findings suggest that short segments of naturalistic clinical dialogue may contain useful acoustic signals for passively screening patients for cognitive impairment.

## References

[1] Alzheimer’s Disease Association. 2019 Alzheimer’s Disease Facts and Figures. 2019. Accessed March 13, 2019. https://www.alz.org/media/Documents/alzheimers-facts-and-figures-2019-r.pdf

[2] Kotagal V, Langa KM, Plassman BL, Factors associated with cognitive evaluations in the United States. Neurology. Jan 6 2015;84(1):64–71. doi:10.1212/wnl.0000000000001096 25428689 PMC4336093

[3] Federman AD, Becker JH, Mindt MR, Cho D, Curtis L, Wisnivesky J. Rates of Undiagnosed Cognitive Impairment and Performance on the Montreal Cognitive Assessment Among Older Adults in Primary Care. J Gen Intern Med. Feb 22 2023;doi:10.1007/s11606-023-08102-w PMC10465418 36814049

[4] Lin JS, O’Connor E, Rossom RC, Perdue LA, Eckstrom E. Screening for cognitive impairment in older adults: A systematic review for the U.S. Preventive Services Task Force. Ann Intern Med. Nov 5 2013;159(9):601–12. doi:10.7326/0003-4819-159-9-201311050-00730 24145578

[5] Chodosh J, Petitti DB, Elliott M, Physician recognition of cognitive impairment: evaluating the need for improvement. J Am Geriatr Soc. Jul 2004;52(7):1051–9. doi:10.1111/j.1532-5415.2004.52301.x 15209641

[6] Bradford A, Kunik ME, Schulz P, Williams SP, Singh H. Missed and delayed diagnosis of dementia in primary care: prevalence and contributing factors. Alzheimer Dis Assoc Disord. Oct-Dec 2009;23(4):306–14. doi:10.1097/WAD.0b013e3181a6bebc 19568149 PMC2787842

[7] Valcour VG, Masaki KH, Curb JD, Blanchette PL. The detection of dementia in the primary care setting. Arch Intern Med. Oct 23 2000;160(19):2964–8. 11041904 10.1001/archinte.160.19.2964

[8] Koch T, Iliffe S. Rapid appraisal of barriers to the diagnosis and management of patients with dementia in primary care: a systematic review. BMC Fam Pract. Jul 1 2010;11:52. doi:10.1186/1471-2296-11-52 20594302 PMC2909966

[9] Hinton L, Franz C, Friend J. Pathways to dementia diagnosis: evidence for cross-ethnic differences. Alzheimer Disease & Associated Disorders. 2004 Jul 1;18(3):134–44 15494619 10.1097/01.wad.0000127444.23312.ff

[10] Owens DK, Davidson KW, Krist AH, Screening for Cognitive Impairment in Older Adults: US Preventive Services Task Force Recommendation Statement. Jama. Feb 25 2020;323(8):757–763. doi:10.1001/jama.2020.0435 32096858

[11] Pinquart M, Sorensen S. Helping caregivers of persons with dementia: which interventions work and how large are their effects? Int Psychogeriatr. Dec 2006;18(4):577–95. doi:10.1017/s1041610206003462 16686964

[12] Pusey H, Richards D. A systematic review of the effectiveness of psychosocial interventions for carers of people with dementia. Aging Ment Health. May 2001;5(2):107–19. 11511058 10.1080/13607860120038302

[13] Cooke DD, McNally L, Mulligan KT, Harrison MJ, Newman SP. Psychosocial interventions for caregivers of people with dementia: a systematic review. Aging Ment Health. May 2001;5(2):120–35. 11511059 10.1080/713650019

[14] Gaugler JE, Kane RL, Kane RA, Newcomer R. Early community-based service utilization and its effects on institutionalization in dementia caregiving. Gerontologist. Apr 2005;45(2):177–85. 15799982 10.1093/geront/45.2.177

[15] Dixon J, Karagiannidou M, Knapp M. The Effectiveness of Advance Care Planning in Improving End-of-Life Outcomes for People With Dementia and Their Carers: A Systematic Review and Critical Discussion. J Pain Symptom Manage. Jan 2018;55(1):132–150.e1. doi:10.1016/j.jpainsymman.2017.04.009 28827062

[16] Clarkson P, Davies L, Jasper R, Loynes N, Challis D. A Systematic Review of the Economic Evidence for Home Support Interventions in Dementia. Value Health. Sep 2017;20(8):1198–1209. doi:10.1016/j.jval.2017.04.004 28964453

[17] Austrom MG, Boustani M, LaMantia MA. Ongoing Medical Management to Maximize Health and Well-being for Persons Living With Dementia. Gerontologist. Jan 18 2018;58(suppl_1):S48–s57. doi:10.1093/geront/gnx147 29361066 PMC5881733

[18] Backhouse A, Ukoumunne OC, Richards DA, McCabe R, Watkins R, Dickens C. The effectiveness of community-based coordinating interventions in dementia care: a meta-analysis and subgroup analysis of intervention components. BMC Health Serv Res. Nov 13 2017;17(1):717. doi:10.1186/s12913-017-2677-2 29132353 PMC5683245

[19] Kok RM, Reynolds CF, 3rd. Management of Depression in Older Adults: A Review. Jama. May 23 2017;317(20):2114–2122. doi:10.1001/jama.2017.5706 28535241

[20] Smith D, Lovell J, Weller C, A systematic review of medication non-adherence in persons with dementia or cognitive impairment. PloS one. 2017;12(2):e0170651. doi:10.1371/journal.pone.0170651 28166234 PMC5293218

[21] Thyrian JR, Hertel J, Wucherer D, Effectiveness and Safety of Dementia Care Management in Primary Care: A Randomized Clinical Trial. JAMA psychiatry. Oct 1 2017;74(10):996–1004. doi:10.1001/jamapsychiatry.2017.2124 28746708 PMC5710469

[22] van Dyck CH, Swanson CJ, Aisen P, Lecanemab in Early Alzheimer’s Disease. N Engl J Med. Jan 5 2023;388(1):9–21. doi:10.1056/NEJMoa2212948 36449413

[23] Congdon EE, Ji C, Tetlow AM, Jiang Y, Sigurdsson EM. Tau-targeting therapies for Alzheimer disease: current status and future directions. Nature reviews Neurology. Oct 24 2023;doi:10.1038/s41582-023-00883-2 PMC10965012 37875627

[24] Athilingam P, Visovsky C, Elliott AF, Rogal PJ. Cognitive screening in persons with chronic diseases in primary care: challenges and recommendations for practice. Am J Alzheimers Dis Other Demen. Sep 2015;30(6):547–58. doi:10.1177/1533317515577127 25794511 PMC10852828

[25] Scott J, Mayo AM. Instruments for detection and screening of cognitive impairment for older adults in primary care settings: A review. Geriatric nursing (New York, NY). May - Jun 2018;39(3):323–329. doi:10.1016/j.gerinurse.2017.11.001 29268944

[26] Weir DR, Wallace RB, Langa KM, Reducing case ascertainment costs in U.S. population studies of Alzheimer’s disease, dementia, and cognitive impairment-Part 1. Alzheimers Dement. Jan 2011;7(1):94–109. doi:10.1016/j.jalz.2010.11.004 21255747 PMC3044596

[27] Barnes DE, Zhou J, Walker RL, Larson EB, Lee SJ, Boscardin WJ, Marcum ZA, Dublin S. Development and Validation of eRADAR: A Tool Using EHR Data to Detect Unrecognized Dementia. J Am Geriatr Soc. 2020 Jan;68(1):103–111. doi: 10.1111/jgs.16182 31612463 PMC7094818

[28] Penfold RB, Carrell DS, Cronkite DJ, Pabiniak C, Dodd T, Glass AM, Johnson E, Thompson E, Arrighi HM, Stang PE. Development of a machine learning model to predict mild cognitive impairment using natural language processing in the absence of screening. BMC Med Inform Decis Mak. 2022 May 12;22(1):129. doi: 10.1186/s12911-022-01864-z 35549702 PMC9097352

[29] Yadgir SR, Engstrom C, Jacobsohn GC, Green RK, Jones CMC, Cushman JT, Caprio TV, Kind AJH, Lohmeier M, Shah MN, Patterson BW. Machine learning-assisted screening for cognitive impairment in the emergency department. J Am Geriatr Soc. 2022 Mar;70(3):831–837. doi: 10.1111/jgs.17491 34643944 PMC8904269

[30] Yim D, Yeo TY, Park MH. Mild cognitive impairment, dementia, and cognitive dysfunction screening using machine learning. J Int Med Res. 2020 Jul;48(7):300060520936881. doi: 10.1177/0300060520936881 32644870 PMC7350047

[31] Schäfer S, Mallick E, Schwed L, König A, Zhao J, Linz N, Bodin TH, Skoog J, Possemis N, Ter Huurne D, Zettergren A, Kern S, Sacuiu S, Ramakers I, Skoog I, Tröger J. Screening for Mild Cognitive Impairment Using a Machine Learning Classifier and the Remote Speech Biomarker for Cognition: Evidence from Two Clinically Relevant Cohorts. J Alzheimers Dis. 2023;91(3):1165–1171. doi: 10.3233/JAD-220762 36565116 PMC9912722

[32] Li Q, Yang X, Xu J, Guo Y, He X, Hu H, Lyu T, Marra D, Miller A, Smith G, DeKosky S, Boyce RD, Schliep K, Shenkman E, Maraganore D, Wu Y, Bian J. Early prediction of Alzheimer’s disease and related dementias using real-world electronic health records. Alzheimers Dement. 2023 Aug;19(8):3506–3518. doi: 10.1002/alz.12967. 36815661 PMC10976442

[33] Kappen M, Vanderhasselt MA, Slavich GM. Speech as a promising biosignal in precision psychiatry. Neurosci Biobehav Rev. 2023 May;148:105121. doi: 10.1016/j.neubiorev.2023.105121 36914080 PMC11219249

[34] de la Fuente Garcia S, Ritchie CW, Luz S. Artificial Intelligence, Speech, and Language Processing Approaches to Monitoring Alzheimer’s Disease: A Systematic Review. J Alzheimers Dis. 2020;78(4):1547–1574. doi: 10.3233/JAD-200888 33185605 PMC7836050

[35] Hoy MB. Alexa, Siri, Cortana, and More: An Introduction to Voice Assistants. Med Ref Serv Q. 2018 Jan-Mar;37(1):81–88. doi: 10.1080/02763869.2018.1404391 29327988

[36] Tao F, Mirheidari B, Pahar M, Young S, Xiao Y, Elghazaly H, Peters F, Illingworth C, Braun D, O’Malley R, Bell S. Early dementia detection using multiple spontaneous speech prompts: The process challenge. ICASSP 2025-2025 IEEE International Conference on Acoustics, Speech and Signal Processing (ICASSP). 2025 Apr 6 (pp. 1–2). IEEE. doi: 10.1109/ICASSP49660.2025.10889017

[37] Zolnoori M, Vergez S, Kostic Z, Jonnalagadda S, V McDonald M, Bowles K, Topaz M Audio Recording Patient-Nurse Verbal Communications in Home Health Care Settings: Pilot Feasibility and Usability Study. JMIR Hum Factors 2022;9(2):e35325. doi: 10.2196/35325 35544296 PMC9133990

[38] Nasreddine ZS, Phillips NA, Bedirian V, The Montreal Cognitive Assessment, MoCA: a brief screening tool for mild cognitive impairment. J Am Geriatr Soc. Apr 2005;53(4):695–9. doi:10.1111/j.1532-5415.2005.53221.x 15817019

[39] Rossetti HC, Lacritz LH, Cullum CM, Weiner MF. Normative data for the Montreal Cognitive Assessment (MoCA) in a population-based sample. Neurology. Sep 27 2011;77(13):1272–5. doi:10.1212/WNL.0b013e318230208a 21917776

[40] Freitas S, Simoes MR, Alves L, Santana I. Montreal cognitive assessment: validation study for mild cognitive impairment and Alzheimer disease. Alzheimer Dis Assoc Disord. Jan-Mar 2013;27(1):37–43. doi:10.1097/WAD.0b013e3182420bfe 22193353

[41] Yadgir SR, Engstrom C, Jacobsohn GC, Machine learning-assisted screening for cognitive impairment in the emergency department. J Am Geriatr Soc. Oct 13 2021;doi:10.1111/jgs.17491 PMC8904269 34643944

[42] Trittschuh EH, Crane PK, Larson EB, Effects of varying diagnostic criteria on prevalence of mild cognitive impairment in a community based sample. J Alzheimers Dis. 2011;25(1):163–73. doi:10.3233/jad-2011-101821 21368379 PMC3146555

[43] Jak AJ, Preis SR, Beiser AS, Neuropsychological Criteria for Mild Cognitive Impairment and Dementia Risk in the Framingham Heart Study. J Int Neuropsychol Soc. Oct 2016;22(9):937–943. doi:10.1017/s1355617716000199 27029348 PMC5045758

[44] Busse A, Hensel A, Gühne U, Angermeyer MC, Riedel-Heller SG. Mild cognitive impairment: long-term course of four clinical subtypes. Neurology. Dec 26 2006;67(12):2176–85. doi:10.1212/01.wnl.0000249117.23318.e1 17190940

[45] Silero Team. Silero VAD: Pre-trained Enterprise-grade Voice Activity Detector (VAD), Number Detector and Language Classifier. GitHub repository. Published 2024. Accessed June 30, 2025. https://github.com/snakers4/silero-vad

[46] Petermann D, Wichern G, Wang ZQ, Le Roux J. The cocktail fork problem: three-stem audio separation for real-world soundtracks. Proceedings of the IEEE International Conference on Acoustics, Speech and Signal Processing (ICASSP). May 23–27, 2022; Singapore. IEEE; 2022:526–530. doi:10.1109/ICASSP43922.2022.9746005

[47] Radford A, Kim JW, Xu T, Brockman G, McLeavey C, Sutskever I. Robust speech recognition via large-scale weak supervision. Proceedings of the International Conference on Machine Learning. July 2023:28492–28518. PMLR. doi:10.48550/arXiv.2212.04356

[48] Agbavor F, Liang H. Multilingual prediction of cognitive impairment with large language models and speech analysis. Brain Sci. 2024;14(12):1292. doi: 10.3390/brainsci14121292 39766491 PMC11674350

[49] Wei-Ning H, Benjamin B, Yao-Hung H, Kushal L, Ruslan S, Abdelrahman M. HuBERT: Self-Supervised Speech Representation Learning by Masked Prediction of Hidden Units. IEEE/ACM Trans. Audio, Speech and Lang. Proc. 29 (2021), 3451–3460. doi:10.1109/TASLP.2021.3122291

[50] Chen M., Miao C., Ma J., Wang S., Xiao J. (2023) Exploring multi-task learning and data augmentation in dementia detection with self-supervised pretrained models. Proc. Interspeech 2023. 5037–5041. doi: 10.21437/Interspeech.2023-1623

[51] Baevski A, Zhou Y, Mohamed A, Auli M. wav2vec 2.0: a framework for self-supervised learning of speech representations. Adv Neural Inf Process Syst. 2020;33:12449–12460. doi: 10.48550/arXiv.2006.11477

[52] Ying Y., Yang T. & Zhou H. Multimodal fusion for alzheimer’s disease recognition. Appl Intell 53, 16029–16040 (2023). doi:10.1007/s10489-022-04255-z

[53] Eyben F, Scherer KR, Schuller BW, Sundberg J, André E, Busso C, Devillers L, Epps J, Laukka P, Narayanan SS, Truong KP. The Geneva minimalistic acoustic parameter set (GeMAPS) for voice research and affective computing. IEEE Trans Affect Comput. 2015;7(2):190–202. doi: 10.1109/TAFFC.2015.2457417

[54] Eyben F, Wöllmer M, Schuller B. openSMILE: the Munich versatile and fast open-source audio feature extractor. In: Proceedings of the 18th ACM International Conference on Multimedia. October 25, 2010:1459–1462. doi:10.1145/1873951.1874246

[55] Luz S., Haider F., Fuente S.d.l., Fromm D., MacWhinney B. (2021) Detecting Cognitive Decline Using Speech Only: The ADReSSo Challenge. Proc. Interspeech 2021, 3780–3784, doi: 10.21437/Interspeech.2021-1220

[56] Dehak N, Dumouchel P, Kenny P. Modeling prosodic features with joint factor analysis for speaker verification. IEEE Trans Audio Speech Lang Process. 2007;15(7):2095–2103. doi: 10.1109/TASL.2007.902758

[57] Vásquez-Correa JC, Arias-Vergara T, Rios-Urrego CD, Schuster M, Rusz J, Orozco-Arroyave JR. Towards an automatic evaluation of the dysarthria level of patients with Parkinson’s disease. J Commun Disord. 2018;76:21–36 30149241 10.1016/j.jcomdis.2018.08.002

[58] Farzana S, Parde N. Towards domain-agnostic and domain-adaptive dementia detection from spoken language. In: Proceedings of the 61st Annual Meeting of the Association for Computational Linguistics (Volume 1: Long Papers). 2023:11965–11978. doi: 10.18653/v1/2023.acl-long.668

[59] Chen T, Guestrin C. XGBoost: a scalable tree boosting system. In: Proceedings of the 22nd ACM SIGKDD International Conference on Knowledge Discovery and Data Mining. August 13, 2016:785–794. doi: 10.1145/2939672.29397

[60] Lee J, Yoon W, Kim S, Kim D, Kim S, So CH, Kang J. BioBERT: a pre-trained biomedical language representation model for biomedical text mining. Bioinformatics. 2020 Feb 15;36(4):1234–1240. doi: 10.1093/bioinformatics/btz682 31501885 PMC7703786

[61] Radivojac P, Clark WT, Oron TR, Schnoes AM, Wittkop T, Sokolov A, Graim K, Funk C, Verspoor K, Ben-Hur A, A large-scale evaluation of computational protein function prediction. Nat Methods. 2013;10(3):221–227. doi: 10.1038/nmeth.2340 23353650 PMC3584181

[62] Lundberg SM, Lee SI. A unified approach to interpreting model predictions. Advances in neural information processing systems. 2017;30. doi: 10.5555/3295222.3295230

[63] Guo T, Li S, Unoki M, Okada S. Investigation of noise-reverberation-robustness of modulation spectral features for speech-emotion recognition. 2022 Asia-Pacific Signal and Information Processing Association Annual Summit and Conference (APSIPA ASC) 2022 Nov 7 (pp. 39–46). IEEE. doi: 10.23919/apsipaasc55919.2022.9980032

[64] Barrera-Altuna B, Lee D, Zarnaz Z, Han J, Kim S. The Interspeech 2024 TAUKADIAL Challenge: Multilingual Mild Cognitive Impairment Detection with Multimodal Approach. group. 2024;25:26. doi: 10.21437/Interspeech.2024-1352

[65] Gao Y, Guo L, Liu H. Leveraging Multimodal Methods and Spontaneous Speech for Alzheimer’s Disease Identification. ICASSP 2025-2025 IEEE International Conference on Acoustics, Speech and Signal Processing (ICASSP) 2025 Apr 6 (pp. 1–2). IEEE. doi: 10.1109/ICASSP49660.2025.

[66] Rektorova I, Mekyska J, Janousova E, Kostalova M, Eliasova I, Mrackova M, Berankova D, Necasova T, Smekal Z, Marecek R. Speech prosody impairment predicts cognitive decline in Parkinson’s disease. Parkinsonism & related disorders. 2016 Aug 1;29:90–5. 10.1016/j.parkreldis.2016.05.018 27237105

[67] Saeedi S, Hetjens S, Grimm MO, Latoszek BB. Acoustic Speech Analysis in Alzheimer’s Disease: A Systematic Review and Meta-Analysis. The Journal of Prevention of Alzheimer’s Disease. 2024 Jun 1;11(6):1789–97. doi: 10.14283/jpad.2024.132 PMC11573841 39559890

[68] Martínez-Nicolás I, Llorente TE, Martínez-Sánchez F, Meilán JJ. Ten years of research on automatic voice and speech analysis of people with Alzheimer’s disease and mild cognitive impairment: a systematic review article. Frontiers in Psychology. 2021 Mar 23;12:620251. doi: 10.3389/fpsyg.2021.620251 33833713 PMC8021952

