# Supplementary material for: Acoustic Analysis of Primary Care Patient-provider Conversations to Screen for Cognitive Impairment
Source: medRxiv. 2025 Dec 29:2025.12.27.25343088. Preprint. [Version 1] doi: 10.64898/2025.12.27.25343088 (PMC12772652; doi:10.64898/2025.12.27.25343088)
Supplement: 1 [file NIHPP2025.12.27.25343088v1-supplement-1.pdf]

medRxiv preprint doi: https://doi.org/10.64898/2025.12.27.25343088; this version posted December 29, 2025. The copyright holder for this preprint (which was not certified by peer review) is the author/funder, who has granted medRxiv a license to display the preprint in perpetuity.

It is made available under a CC-BY 4.0 International license .

SUPPLEMENTARY MATERIALS

Supplemental Table 1. Mount Sinai and Northwestern Audio Data

			(N)	Mean Audio Duration (Minutes)	Std Dev of Audio (Minutes)
MSSM Total			787	29.63	13.04
	HC		624 (79.3%)	29.24	12.78
	CI		163 (20.7%)	31.12	13.94
	Male		432 (54.9%)	31.28	13.73
		HC	332 (42.2%)	30.81	13.45
		CI	100 (12.7%)	32.82	14.59
	Female		355 (45.1%)	27.63	11.87
		HC	292 (37.1%)	27.46	11.75
		CI	63 (8.0%)	28.43	12.50
NU Total			179	23.65	9.71
	HC		146 (81.6%)	24.24	9.53
	CI		33 (18.4%)	21.04	10.24
	Male		98 (54.7%)	22.91	9.81
		HC	82 (45.8%)	23.12	9.43
		CI	16 (8.9%)	21.86	11.89
	Female		81 (45.3%)	24.55	9.57
		HC	64 (35.8%)	25.68	9.54
		CI	17 (9.5%)	20.28	8.70

medRxiv preprint doi: https://doi.org/10.64898/2025.12.27.25343088; this version posted December 29, 2025. The copyright holder for this preprint (which was not certified by peer review) is the author/funder, who has granted medRxiv a license to display the preprint in perpetuity.

It is made available under a CC-BY 4.0 International license .

Supplemental Section 1. Acoustic Feature Extraction

Whisper Embeddings: We used the audio encoder from OpenAI's Whisper large-v2 model to derive high-level acoustic embeddings from each 30-second segment.[47] The model's multilingual training and robustness to real-world noise make it well-suited for clinical settings.[48]

HuBERT Embeddings: We applied the Hidden-Unit BERT (HuBERT) Base model (finetuned on LibriSpeech) to generate self-supervised acoustic representations.[49] HuBERT clusters latent speech units and uses them to predict masked inputs, capturing subphonemic patterns relevant for cognitive and affective speech analysis.[50]

Wav2Vec 2.0 Embeddings: We extracted representations from the Wav2Vec 2.0 Base Robust model (W2V2).[51] Like HuBERT, W2V2 learns contextual embeddings using contrastive predictive coding. These embeddings offer complementary information regarding speech fluency, articulation, and rhythm.[52]

eGeMAPS Acoustic Features: We computed the Extended Geneva Minimalistic Acoustic Parameter Set (eGeMAPS) using the openSMILE toolkit.[53-54] eGeMAPS includes 88 low-level descriptors such as jitter, shimmer, formant frequencies, MFCCs, and spectral slope. These features have been linked to neurological and psychiatric status.[55]

medRxiv preprint doi: https://doi.org/10.64898/2025.12.27.25343088; this version posted December 29, 2025. The copyright holder for this preprint (which was not certified by peer review) is the author/funder, who has granted medRxiv a license to display the preprint in perpetuity.

It is made available under a CC-BY 4.0 International license .

Prosodic Features: Prosodic cues including pitch (F0), speaking rate, pause duration, and voicing probability were extracted using the DisVoice toolkit.[56-57] These features quantify the suprasegmental aspects of speech and have been shown to vary in cognitive decline.[58]
